# History Matters in Solid-State Hydrogen Storage: Hidden State Variables and Pathway-Dependent Reactivity in Mg-Based Hydrides

**DOI:** 10.3390/molecules31111982

**Published:** 2026-06-05

**Authors:** Chen Chen, Quanhui Hou, Liangjuan Gao, Zhao Ding

**Affiliations:** 1 Department of Mechanics, Jinzhong University, Jinzhong 030606, China; chenchentgzy@163.com; 2 School of Automotive Engineering, Yancheng Institute of Technology, Yancheng 224051, China; hqhdyx66@ycit.edu.cn; 3 College of Materials Science and Engineering, Sichuan University, Chengdu 610065, China; lgao87@scu.edu.cn; 4 College of Materials Science and Engineering, National Engineering Research Center for Magnesium Alloys, Chongqing University, Chongqing 400044, China

**Keywords:** solid-state hydrogen storage, magnesium hydride, hidden state variables, metastability, pathway-dependent reactivity, interface engineering, catalyst state, hydrogen sorption mechanism

## Abstract

Magnesium-based hydrides remain among the most intensively studied solid-state hydrogen storage materials because they combine high theoretical hydrogen capacity, elemental abundance, and relatively low cost. Yet their practical behavior often varies far more strongly than nominal composition alone would suggest. Materials described under similar chemical labels may show markedly different activation profiles, sorption kinetics, reversible capacities, and cycling responses, even when they appear compositionally comparable. This Perspective argues that such discrepancies are best understood by recognizing that Mg-based hydrogen storage materials are not fully defined by composition, catalyst identity, and equilibrium thermodynamics alone. Instead, they react from historically written states produced by synthesis, activation, and cycling. These histories generate hidden state variables, including defects, residual strain, metastable structural motifs, interfacial topology, and catalyst transformation states, that reshape the operative hydrogen sorption pathway. The discussion therefore moves from a conventional composition-centered view toward a pathway-centered interpretation of reactivity. First, it examines how hidden state variables are written into Mg-based materials through processing, activation, and repeated use. It then shows how metastability serves as the structural bridge that allows these variables to persist into the reaction window. On that basis, the article argues that hydrogen sorption in Mg-based hydrides is fundamentally pathway-dependent, with history influencing hydrogen entry, transport-network selection, interfacial route construction, and pathway evolution during cycling. This perspective also provides a more coherent explanation for the long-standing reproducibility problem in the field, which is reinterpreted here as a pathway-mismatch problem arising from comparisons among historically different reactive states. Finally, a metadata-aware, pathway-aware, and boundary-aware design framework is proposed as a more realistic basis for cumulative materials development. From this viewpoint, the future of Mg-based solid-state hydrogen storage depends not only on better compositions, but on better-defined, better-constructed, and better-preserved reactive pathways under clearly specified internal and external constraints.

## 1. Introduction

Hydrogen storage remains the principal obstacle separating the conceptual appeal of hydrogen as a clean energy carrier from its widespread technological implementation. Among the available storage routes, solid-state hydrogen storage has long attracted attention because it offers a fundamentally different balance among safety, volumetric density, reversibility, and integration potential from those available in compressed-gas and liquefied-hydrogen systems [1,2,3]. Within this broader field, magnesium-based materials occupy a particularly important and somewhat paradoxical position. Magnesium is abundant, inexpensive, relatively light, and capable, in principle, of storing hydrogen at levels that remain attractive even when judged against contemporary performance criteria. The theoretical capacity of MgH_2_, approximately 7.6 wt.%, continues to justify sustained interest in Mg-based systems as candidate materials for practical hydrogen storage [4]. At the same time, however, the same material family that appears so compelling in gravimetric terms is also burdened by the characteristics that have limited its wider application for decades, namely strong Mg–H bonding, high thermodynamic stability, sluggish hydrogen sorption kinetics, poor low-temperature reactivity, and substantial structural evolution during repeated hydrogenation and dehydrogenation. The history of Mg-based hydrogen storage is therefore not simply the story of a promising material, but the story of repeated attempts to retain its attractive hydrogen capacity while mitigating the kinetic and structural penalties imposed by hydride stability [2,3,4,5].

The standard language used to discuss this problem has become highly familiar. Most descriptions of Mg-based hydrogen storage still begin from the triad of nominal composition, catalyst identity, and equilibrium thermodynamics. In this framework, the key questions are usually posed in straightforward form. Which additive lowers the dehydrogenation temperature? Which synthesis route improves hydrogen absorption and desorption rates? Which structural modification enhances reversibility or suppresses degradation during cycling? This way of asking the problem has been productive, and it has motivated a large body of valuable work on transition-metal additions, oxide catalysts, nanostructuring, severe plastic deformation, nanoconfinement, precursor chemistry, and reactive composite design [6,7,8,9,10]. Yet the growth of that literature has also made a deeper issue increasingly visible. Mg or MgH_2_-based materials described under very similar chemical labels often behave in markedly different ways. Samples identified as ball-milled MgH_2_ with the same additive and loading may show different activation profiles, different onset temperatures, different sorption rates, different reversible capacities, and different cycle evolution. These discrepancies are commonly attributed to particle size, catalyst dispersion, defect density, or surface oxidation. While such explanations are not wrong, they are often too local and too fragmented to account for the broader pattern. What is still too rarely asked in an explicit and systematic way is whether the materials being compared were ever in the same reactive state at all [6,7,8,9,10].

That question matters because the experimentally relevant material in Mg-based hydrogen storage is not fully specified by composition alone. The solid that enters a Sieverts apparatus or a thermal-desorption experiment is never just a formula. It is the consequence of prior processing, prior exposure, prior activation, and prior structural evolution. Defect density, retained strain, metastable phase fraction, near-surface chemistry, catalyst transformation state, interfacial connectivity, crack history, and local transport topology all contribute to the condition from which hydrogen sorption begins [11,12,13,14]. Some of these features are partially visible in conventional structural characterization. Others remain hidden unless they are targeted deliberately. Yet many of them are mechanistically decisive. A defect-rich near-surface region, a retained nanocrystalline or partially amorphous state, a catalyst that becomes active only after in situ transformation, or a buried heterointerface that serves as a transport shortcut can each alter not only how rapidly hydrogen moves, but how hydrogen enters, where it nucleates, which interfaces become reactive, and through which route the overall transformation proceeds. Once these features are recognized as part of the material rather than as context surrounding the material, the interpretive framework of Mg-based hydrogen storage changes in a fundamental way [13,14].

The present Perspective is built around a stronger and more specific proposition than the familiar statement that processing matters. The central claim is that material history writes hidden state variables into Mg-based hydrides, and those hidden state variables reshape the hydrogen sorption pathway itself [13,14]. The primary question is therefore not only why one material reacts faster than another, but why nominally similar materials may begin reaction from different surface conditions, use different internal transport networks, activate different interfacial chemistries, and evolve through different phase-transformation routes. This is a pathway problem rather than merely a rate problem. It is also a more faithful reading of a literature in which “improved kinetics” often turns out, on closer inspection, to mean that the route of hydrogen entry, migration, nucleation, decomposition, or recombination has been altered by a historically constructed structural state. Once the problem is formulated in this way, a number of recurring features of the field become easier to understand, including the strong sensitivity of Mg-based systems to activation procedure, the repeated appearance of metastable high-performance states, the fragility of many literature comparisons across laboratories, and the difficulty of distinguishing truly transferable strategies from those that depend on narrow historical conditions.

**Figure 1:**
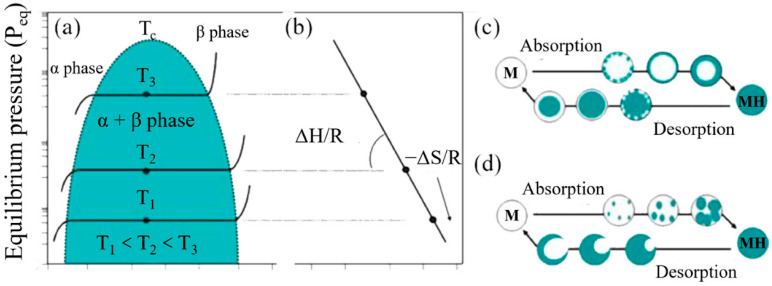
Thermodynamic and kinetic background of hydrogen storage in magnesium-based systems. (**a**) Pressure–composition isotherm of hydrogen–metal systems. (**b**) Van’t Hoff relation for the hydriding/dehydriding reaction. (**c**) Schematic hydrogen absorption/desorption behavior in magnesium under relatively high temperature and pressure, where rapid surface hydride/metal shell formation can impede further hydrogen diffusion. (**d**) Schematic hydrogen absorption/desorption behavior under relatively low temperature and pressure, where phase nucleation and inward diffusion proceed more gradually. Adapted from Ref. [5].

The conventional composition-centered picture remains indispensable, and it is summarized in Figure 1, which places pressure–composition behavior, Van’t Hoff thermodynamics, and the familiar shell-growth description of Mg hydrogenation and dehydrogenation at the front of the discussion [5]. That picture continues to define the baseline from which most work in the field begins. Yet it does not, by itself, explain why apparently similar materials can occupy very different reactive conditions or follow distinctly different sorption routes. The present article therefore begins from the conventional framing but does not end there. It asks what lies between nominal composition and observed sorption behavior, and it argues that the missing layer is a historically encoded set of hidden state variables that govern the operative pathway of hydrogen exchange.

The purpose of the following sections is not to replace thermodynamics, catalyst chemistry, or nanostructure with a new master concept, but to reinterpret them through a state- and pathway-based lens. Section 2 shows how hidden state variables are written into the material through synthesis, activation, and cycling. Section 3 explains why these historically written states remain relevant by being retained, at least partially, in metastable form. Section 4 then argues that the practical consequence of this retention is pathway-dependent reactivity. Section 5 explains why this mechanistic picture helps clarify the persistent reproducibility problem in the field. Section 6 finally proposes a metadata-aware, pathway-aware, and boundary-aware design framework as a more realistic basis for cumulative progress in Mg-based solid-state hydrogen storage.

## 2. Hidden State Variables Are Written by History

The hidden state variables that govern hydrogen sorption in Mg-based hydrides do not appear randomly. They are written into the material in a historically ordered manner, first through synthesis, then through activation, and finally through continued cycling. Each of these stages is often described in the literature as though it were mainly procedural: synthesis is the route by which a target material is prepared, activation is the practical step that precedes reproducible testing, and cycling is the means by which stability is evaluated. These descriptions are not incorrect, but they are mechanistically incomplete. From the standpoint of reactivity, synthesis is the stage at which the first reactive state is written, activation is the stage at which that latent state is converted into an operative one, and cycling is the stage at which the state is repeatedly modified and partially rewritten. The material relevant to hydrogen sorption is therefore not merely the product of synthesis. It is the cumulative result of state formation across these linked histories [5,6].

Synthesis history writes the first layer of hidden state variables because every preparation route creates its own defect structure, interfacial topology, surface condition, and local energetic landscape. Mechanical milling remains the clearest example. High-energy ball milling does much more than reduce particle size [6,7,8,9,10,11,12]. It repeatedly fractures and rewelds particles, stores strain, generates dislocations, creates grain boundaries, increases fresh surface area, and in some systems drives partial amorphization or extensive nanocrystallization. These consequences are not secondary to the chemistry of MgH_2_. They define the initial condition from which hydrogen sorption later begins. A ball-milled MgH_2_ powder is not simply coarse MgH_2_ scaled down. It is a non-equilibrium reactive state with a different defect content, a different boundary density, a different near-surface disorder profile, and a different local transport architecture [6,7,8,9,10,11,12].

The same conclusion extends beyond ball milling. Ultrafine nanocomposites generated by ultrahigh-energy, high-pressure milling do not merely intensify a familiar route, they write a more extreme version of refinement, interfacial continuity, and defect retention into the hydride [11,12]. Gas-phase condensation and in situ hydrogenation routes create still another type of reactive state, one shaped by a different surface history, a different nucleation environment, and a different balance between structural integrity and local strain than those typical of top-down mechanical routes [13,14]. Organometallic and ligand-mediated routes make the point even more sharply because they enable low-temperature or solution-based pathways to nanostructured Mg or MgH_2_ states that are not simply reduced-size analogues of milled materials. In these cases, the operative difference lies not only in particle dimensions but in how the particle was written into existence, what its initial surface environment was, how defects or protected shells were retained, and whether metastable or otherwise unusual structural motifs became accessible during formation. Two materials can therefore look similar at the level of nominal size or even nominal composition while still entering hydrogen sorption from significantly different reactive states because their synthetic histories were not equivalent [13,14].

**Figure 2:**
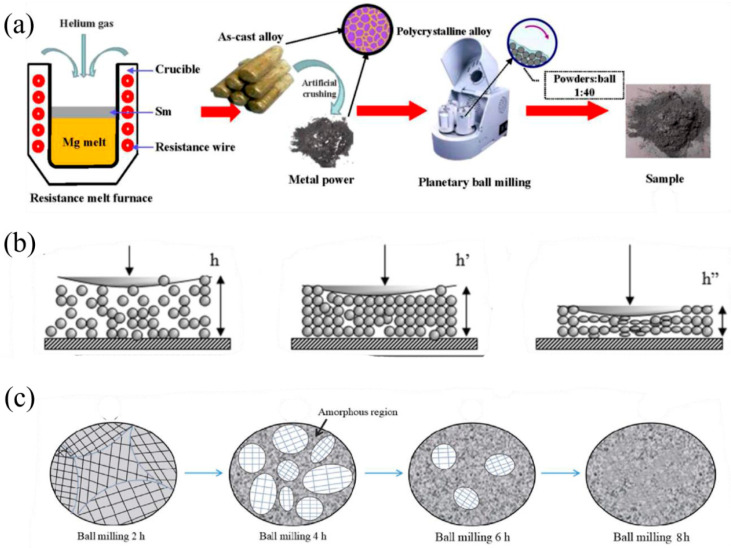
Non-equilibrium state writing during high-energy preparation of Mg-based hydrides. (**a**) Schematic preparation route showing melting/casting, powder production, and subsequent planetary ball milling as a representative high-energy processing sequence for writing the initial reactive state. (**b**) Schematic evolution of powder-bed packing and compression state during milling, illustrating how repeated impact and densification progressively alter particle contact, local constraint, and interfacial continuity. (**c**) Schematic microstructural evolution during prolonged milling, from coarse polycrystalline starting particles to refined, defect-rich, and increasingly homogenized nanostructured states. Together, these changes write the first reactive condition of the material through fracture–welding events, strain accumulation, nanocrystallization, and partial amorphization. Adapted from Ref. [15].

The origin of such hidden state variables is summarized in Figure 2, which should be read not as a general processing overview but as a statement of mechanism [11,12]. High-energy preparation does not simply prepare composition, it writes the first reactive condition of the material. Defects, strain, nanocrystallinity, partial amorphization, and non-equilibrium interfacial arrangements are not by-products that happen to accompany a preparation route. They are the structural and energetic contents of the state that route produces.

Activation writes the second layer of hidden state variables because the as-prepared material is often not yet the one that actually governs the measured sorption route. In Mg-based systems, the initial hydrogenation and dehydrogenation event frequently acts as a state-selection step. Surface oxide or hydroxide layers may be disrupted, partially removed, or bypassed. Additives that were introduced as oxides, nanoparticles, or precursor compounds may transform into different catalytic states. Wetting between additive and host may improve. Buried interfaces may become accessible. Near-surface regions may become enriched in hydrogen-accessible defects or activated exchange sites. In this sense, activation is not simply a practical prelude to the main experiment. It is the stage at which a latent state becomes an operative one.

This point is particularly important in catalyst-containing MgH_2_ systems. The active state of Ni-containing, Nb_2_O_5_-containing, or other catalyst-modified hydrides often cannot be read directly from the as-introduced additive [8,9,10]. What matters is the interfacial configuration and catalytic identity that exist after hydrogen exposure, not before it. Once activation has transformed the near-surface structure and the catalyst–host contact, the reactive material is no longer adequately represented by the as-synthesized description alone [8,9,10]. This is why two nominally identical catalyst-containing samples may behave quite differently if their activation procedures differ, even modestly. The first cycle does not simply reveal pre-existing performance. It helps create it.

Cycling writes the third layer of hidden state variables and, in many cases, the most persistent one. Repeated hydrogen absorption and desorption do not act on a fixed material. They continually reshape the material through volume change, crack formation, boundary motion, local stress accumulation, grain rearrangement, coarsening, catalyst redistribution, and interface reconstruction [13,14]. A sample after ten or fifty cycles is therefore not merely an older version of the starting material. It is a different structural state assembled through repeated chemo-mechanical transformation. In some cases, this history may initially improve reactivity by exposing fresh surfaces or activating favorable transport pathways. In others, it may degrade the route by promoting coarsening, contact loss, or structural relaxation of previously favorable metastable states. What matters for the present Perspective is that cycling is not a passive durability test performed on an unchanging pathway. It is itself a mechanism of state formation.

Once synthesis, activation, and cycling are viewed together, the phrase “hidden state variable” becomes far less metaphorical. These variables are simply the retained consequences of real physical events: fracture, welding, strain accumulation, defect creation, partial amorphization, precursor conversion, interface formation, catalyst transformation, crack evolution, and structural relaxation. They are called hidden only because they are often absent from the shorthand by which materials are named and compared. At this point, the term hidden state variable should no longer remain a conceptual label. In Mg-based hydrogen storage, these variables can be defined more operationally according to when they are written, how they can be measured, and how they are likely to alter hydrogen sorption behavior [13,14]. This step is necessary because many of the features discussed above—defect density, retained strain, catalyst transformation state, interfacial accessibility, and cycling-induced structural evolution—are physically real, yet they often remain absent from the shorthand by which materials are named and compared. Table 1 therefore translates the idea of hidden state variables into a minimum characterization and reporting framework. Rather than treating history as background context, the table identifies which state-sensitive variables are most likely to control hydrogen sorption, when they are introduced, how they may be probed experimentally, and what should be minimally reported if different studies are to remain mechanistically comparable.

Read in this way, hidden state variables are not vague contextual factors but retained consequences of real physical events, including fracture and rewelding during milling, catalyst-state conversion during activation, surface reconstruction, interface redistribution, and cycling-induced microstructural evolution. Their mechanistic importance, however, does not follow from their existence alone. It depends on whether these historically written states remain sufficiently preserved to influence the subsequent hydrogen sorption event. That persistence problem leads directly to metastability. If history were erased as soon as hydrogen exchange began, hidden state variables would matter far less. It is because non-equilibrium structural and interfacial conditions can persist into the reaction window that material history becomes chemically consequential [13,14]. For that reason, metastability forms the necessary bridge between state writing and pathway selection, and it is the natural subject of the next section.

## 3. Metastability as the Bridge Between History and Reactivity

History would matter much less if the structural consequences of synthesis and activation vanished immediately once hydrogen exchange began. The reason they remain important is that Mg-based hydrides often preserve prior processing in the form of metastable states. Metastability is therefore not a secondary topic inserted between synthesis and kinetics for completeness. It is the bridge through which hidden state variables persist into the operative sorption event. In Mg-based systems, where hydrogen exchange is strongly sensitive to defect structure, surface condition, local stress, and interface geometry, this persistence is not incidental. It is frequently the very reason that one material follows one route while another, nominally similar material follows another [15].

The equilibrium thermodynamics of MgH_2_ still provide the essential reference point, but much of the high-performance Mg-based hydrogen storage literature concerns materials that are not reacting from a fully relaxed bulk state [16,17,18,19,20,21,22]. They are nanocrystalline, defect-rich, strained, constrained, interface-dominated, capped, confined, or polymorphically distinct. The significance of metastability in such systems lies not simply in the existence of non-equilibrium structure, but in the retention of that structure long enough to influence hydrogen sorption on the timescale of experiment. A metastable state preserves an altered energetic landscape. It can lower local barriers, shift preferred nucleation sites, redistribute transport pathways, or retain interfacial conditions that would not exist in a relaxed bulk analogue. Once such a state survives into the relevant temperature and pressure window, it becomes part of the reaction environment rather than a historical footnote.

This is already evident in some of the foundational work on nanoscale Mg and MgH_2_. Quantum chemical studies on Mg clusters showed that reducing the system to sufficiently small dimensions changes the energetic landscape of hydrogen storage in ways that are not reducible to bulk expectations [16]. That work did not simply demonstrate a generic size effect, it indicated that the Mg–H system could occupy local energetic conditions in which hydrogen affinity and phase stability were meaningfully altered. Experimental work on magnesium nanowires reinforced this conclusion by showing that dramatically improved absorption and desorption kinetics were linked not merely to shortened diffusion lengths, but to a structurally distinct reactive environment defined by geometry, surface contribution, and local stress accommodation [17]. Nanoconfined MgH_2_ provided a further example, because confining hydride nanoclusters within nanoporous scaffold materials alters not only morphology but also the interfacial and energetic conditions under which sorption occurs [18]. These systems make the same basic point from different directions: once the material is prevented from immediately relaxing into a bulk-like state, history remains present in the reaction window.

The argument becomes even stronger when one considers identifiable metastable hydride states. The existence of γ-MgH_2_ is especially important because it demonstrates directly that MgH_2_ is not limited to one structurally uniform hydride state [19]. Once a distinct polymorph is admitted into the discussion, the larger implication follows: the Mg–H system can be trapped in local minima whose reactivity need not mirror that of conventional bulk MgH_2_. More recent work on precursor-mediated synthesis and protected nanostructures strengthens the same interpretation from a different direction. Superior MgH_2_ nanostructures produced by Grignard-based routes and colloidal or capped Mg systems are not simply examples of elegant synthesis [20]. They show that history can preserve chemically and structurally unusual forms of Mg or MgH_2_ long enough for these forms to govern the actual sorption event. Deformation-assisted routes tell a similar story, because cold rolling and short-time high-energy milling can create hydride states with retained strain, enhanced boundary density, and altered phase constitution that do not disappear immediately upon heating or hydrogen exposure [21,22]. The identification of additional metastable MgH_2_ states under severe mechanical treatment further broadens this picture. These materials are not all the same kind of metastable state, but that is precisely the point. Metastability is not one phenomenon in Mg-based hydrogen storage. It is a general mechanism by which material history remains chemically consequential.

If activated interfaces define where hydrogen first enters a Mg-based material, then the next decisive question is what happens after that entry barrier has been crossed. At this stage, alloying should no longer be interpreted merely as compositional adjustment or as a convenient route to thermodynamic destabilization. Its deeper significance lies in the fact that it reorganizes internal phase relations and thereby reshapes the pathways through which hydrogen is accommodated, transferred, and released. In other words, alloying matters not simply because it changes what phases may exist, but because it changes how those phases are connected during hydrogenation and dehydrogenation. For this reason, alloying in Mg-based hydrogen storage is best understood here as phase-network engineering.

**Figure 3:**
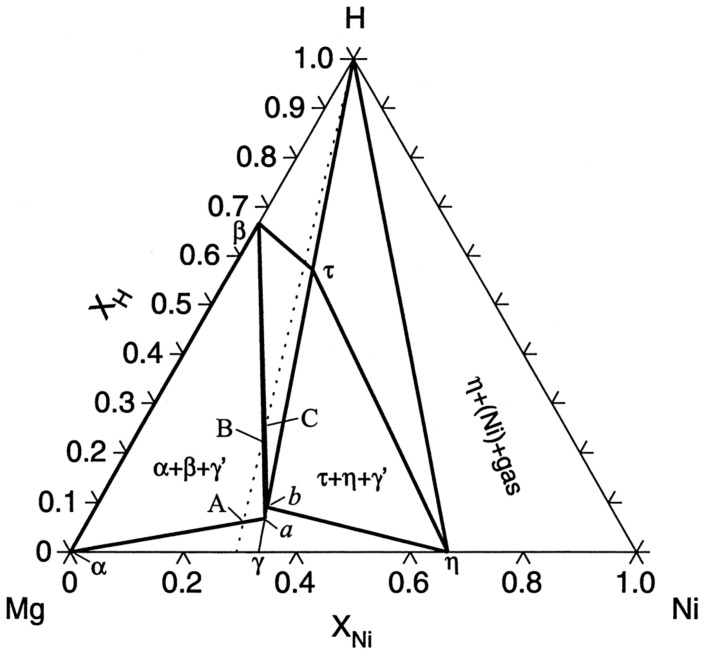
Ternary phase diagram of the Mg–Ni–H system, showing the phase relations relevant to hydrogen absorption and desorption in alloyed Mg–Ni materials. The diagram is used here as a phase-network map, illustrating how alloying reorganizes the internal phase connectivity through which hydrogen is redistributed during sorption. Adapted from Ref. [27].

This perspective becomes especially clear in the Mg–Ni–H system [23,24,25,26]. Once nickel is introduced into magnesium, the hydrogen-storage problem can no longer be described as a simple extension of pure Mg/MgH_2_ chemistry, because the relevant phase space now includes Mg_2_Ni and its hydride Mg_2_NiH_4_, each with distinct thermodynamic and kinetic characteristics. The importance of the Mg–Ni–H ternary phase diagram therefore extends beyond equilibrium description. As shown in Figure 3, the diagram can be read not only as a thermodynamic map, but also as a phase-network map, because it defines the phase fields and phase connections through which hydrogen absorption and desorption must proceed in alloyed Mg–Ni materials. The α and β regions, the two-phase coexistence fields, and the indicated absorption/desorption paths are not merely descriptive labels attached to a ternary system. They define the possible routes by which hydrogen can move between metallic and hydride-bearing environments. In this sense, the diagram helps make visible an internal transport landscape rather than only an equilibrium relation.

Once the ternary system is interpreted this way, several otherwise disconnected observations in Mg-based alloy research become easier to unify. The usefulness of nickel is not exhausted by the statement that Mg_2_NiH_4_ can display more favorable sorption behavior than MgH_2_. What matters more fundamentally is that the formation of Mg_2_Ni/Mg_2_NiH_4_ introduces an additional phase-connected route into the system [23,24,25,26]. Hydrogen can now move through a phase network that is structurally and thermodynamically different from the one available in pure magnesium. The same logic applies more broadly to alloying in Mg-based systems. The advantage of a given alloying element should not be judged only by whether it introduces an intermediate phase or lowers a characteristic desorption temperature. Its importance depends on whether it reorganizes the internal phase landscape in a way that improves continuity between hydrogen entry, accommodation, redistribution, and release.

This viewpoint also clarifies why alloying effects often appear strongly system-specific. The same alloying element may play very different roles depending on whether it enters solid solution, promotes an intermetallic phase, stabilizes a ternary hydride, alters the morphology of phase boundaries, or changes the transport role of pre-existing interfaces [24,25,26]. This is one reason why direct comparison among alloying strategies can be misleading when reduced to one descriptor such as plateau pressure or apparent activation energy. The more relevant mechanistic question is whether the alloyed system offers a better-connected route for hydrogen movement through the material body. From this perspective, the field is not simply searching for the “best alloying element.” It is searching for phase architectures in which interfacial exchange, bulk transport, and reversible transformation remain more effectively coupled. That is the essence of phase-network engineering [27].

The implication for the present article is straightforward. Interface activation, discussed in Section 2, is necessary because hydrogen must first gain entry into the material. But activated entry sites alone do not guarantee high-performance storage if hydrogen then encounters a disconnected or unfavorable phase environment. Alloying becomes important at this point because it determines whether the internal material body offers a phase-connected route or a fragmented one. Yet even favorable phase relations are not always sufficient. A transport route that is thermodynamically and phase-wise accessible may still be too localized, too fragile, or too poorly distributed to sustain efficient hydrogen exchange across the whole material. This is precisely the point at which more elaborate architectures become relevant. Once hydrogen transport is viewed as a problem of continuity across scales, the next logical step is to ask how porous hosts, scaffold-like secondary phases, and hierarchically organized microstructures can amplify and stabilize transport beyond what alloying alone can accomplish.

## 4. Reaction Pathways Are History-Dependent

### 4.1. Pathway Selection Begins at the Surface

Once hidden state variables and metastably retained reactive states are taken seriously, hydrogen sorption in Mg-based systems can no longer be described as though every sample begins from an equivalent surface condition. The reaction always starts at the gas–solid interface, and for Mg-based materials, that interface is rarely a simple metallic or hydride surface. It is more often a boundary region shaped by prior fracture, oxidation, catalyst deposition, segregation, defect retention, surface relaxation, or earlier cycling. Under those conditions, the first decisive event in sorption is not the same process happening at different speeds. It may be a qualitatively different process. In one material, hydrogen uptake may begin through direct dissociative activation at favorable defect-rich or catalyst-assisted sites. In another, it may be delayed or redirected by oxide rupture, local stress release, or penetration through a passivating near-surface layer. This distinction matters because, in Mg-based hydrides, the early interfacial step influences the later geometry of the transformation front, the accessibility of the interior, and the route by which the material proceeds toward hydride growth or decomposition.

**Figure 4:**
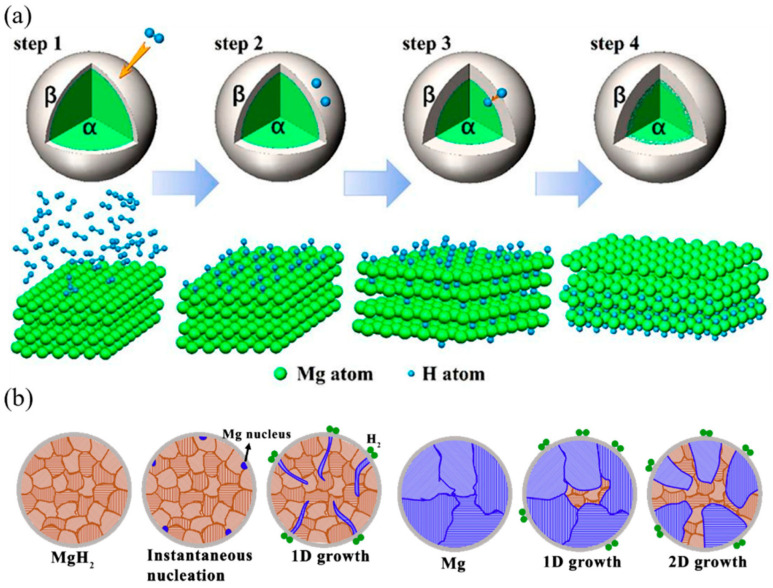
Surface reaction and crystallite-evolution pathways involved in hydrogen sorption in magnesium-based systems. (**a**) Schematic representation of surface hydrogen adsorption, dissociation, inward transport, and hydride growth during Mg hydrogenation. (**b**) Schematic evolution of Mg crystallites during MgH_2_ dehydrogenation, including Mg nucleation and subsequent one-dimensional and two-dimensional growth. Adapted from Refs. [28,29].

The classical kinetic description of Mg hydrogenation and dehydrogenation remains valuable precisely because it clarifies what is being reinterpreted rather than discarded [28,29,30]. Surface adsorption, dissociation, chemisorption, diffusion, and hydride growth still define the broad logic of the process. Likewise, Mg crystallite evolution remains part of the dehydrogenation route rather than a mere morphological aftereffect. These coupled surface-reaction and crystallite-evolution processes are schematically summarized in Figure 4. Direct mechanistic studies of hydrogen release from MgH_2_ further reinforce the conclusion that decomposition is tied to specific structural rearrangements rather than to a featureless bulk response. What changes in the present Perspective is the assumption that these steps always arrange themselves in the same way. They do not. Once the surface has been historically conditioned, pathway selection has already begun.

### 4.2. History Determines Which Transport Network Hydrogen Uses

After hydrogen has crossed the interfacial barrier, history continues to matter because transport inside Mg-based materials does not proceed through a single universal channel. The familiar language of enhanced or sluggish diffusion is often too coarse to capture what is actually happening. In a historically conditioned hydride, hydrogen may move predominantly through the lattice, through grain boundaries, along defects, across interfaces, through crack-assisted shortcuts, or through combinations of these routes that depend on the structural state written into the material. Which of these channels dominates is not a fixed property of the formula. It is a property of the state from which the material reacts.

This is where the relevance of hidden state variables becomes especially clear. Interface-rich biphasic nanoparticles demonstrate that buried interfaces can act as transport-promoting regions that are absent in more relaxed materials [31]. Studies on defect-governed absorption show that crystalline defects can strongly change hydrogen-uptake behavior by altering the accessibility of low-energy entry and migration routes [32]. Vacancy-defective MgH_2_ surfaces reveal an even finer-scale version of the same principle, namely that local dehydrogenation energetics can be changed through defect structures that simultaneously influence surface chemistry and transport topology [33]. Such observations should not be interpreted merely as evidence that defects or interfaces “help.” Their stronger significance is that they define which network hydrogen is actually able to use. Under one historical condition, hydrogen may be constrained by a shell-growth geometry and limited by relatively classical transport. Under another, it may bypass those constraints through defect-enabled or interface-assisted channels. Once this possibility is recognized, many of the broad differences described in the literature as “improved kinetics” can be reformulated more precisely as changes in transport-network selection.

### 4.3. Interfaces and Catalyst States Create Alternative Reaction Routes

The strongest evidence that history rewrites pathway rather than merely rate emerges when catalyst-containing and interface-rich systems are considered. In the traditional composition-centered language, a catalyst is usually introduced as an additive that lowers the barrier of a reaction whose basic route remains unchanged. In Mg-based hydrides, that interpretation is often incomplete. What matters is not only which catalytic species is present, but what state that catalyst reaches after processing and activation. Microscale and nanoscale Ni do not behave identically simply because one offers more surface area than the other [34,35]. They create different interfacial topologies with MgH_2_ and therefore different distributions of active junctions through which hydrogen can enter or leave the hydride. Electronic-structure analysis further shows that transition-metal addition can modify local charge density and weaken Mg–H bonding in specific interfacial environments rather than uniformly throughout the material. Once framed in these terms, catalytic action is no longer a generic lowering of a single barrier. It becomes a way of constructing a different local route through which hydrogen exchange can occur.

**Figure 5:**
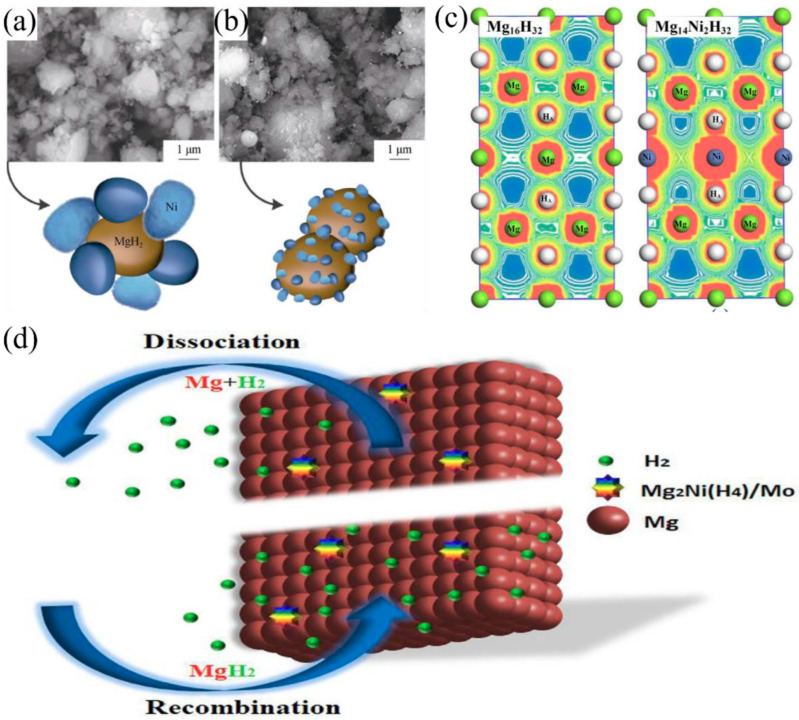
Catalyst-contact geometries, local electronic-structure modulation, and in situ evolution of oxide-derived catalytic states in Mg-based hydrides. (**a**) Contact configuration between microscale Ni and MgH_2_. (**b**) Contact configuration between nanoscale Ni and MgH_2_, showing increased interfacial contact density. (**c**) Transition-metal-induced local electronic-structure modulation in MgH_2_. (**d**) In situ formation of Mg_2_Ni/Mg_2_NiH_4_- and Mo-containing active domains that promote reversible hydrogen dissociation and recombination. Adapted from Ref. [15].

The point becomes even stronger in oxide-derived systems. In NiO/NiMoO_4_-modified MgH_2_, the catalytically relevant state is not simply the oxide additive as introduced [36,37,38]. It is the interfacial state generated in situ during hydrogenation and dehydrogenation, where Mg_2_Ni/Mg_2_NiH_4_- and Mo-containing active domains emerge and collectively produce a hydrogen-pump-like route for reversible exchange. This is an especially important case because it illustrates the difference between catalyst identity and catalyst state. These representative catalyst-contact geometries, transition-metal-induced electronic effects, and oxide-derived in situ catalytic states are summarized in Figure 5. The material is not merely “oxide-doped MgH_2_.” It is a historically evolved interfacial system that acquires access to a route unavailable in the untransformed state. The same reasoning applies to reactive composite systems [39,40,41]. In MgH_2_–LiBH_4_, reversible behavior and improved kinetics arise from the existence of a particular interfacial route involving product formation such as MgB_2_ and LiH, and the accessibility of that route depends strongly on how the interfacial architecture was written into the composite during preparation. Aerosol-assisted or nanostructure-mediated processing does not merely improve mixing. It determines whether the route exists in a practical sense. This is why catalysts and interfaces in Mg-based hydrogen storage should not be treated as separate auxiliary modifiers. They are route-generating features of historically conditioned reactive states.

### 4.4. The Pathway Itself Evolves During Cycling

If the sorption route depends on reactive state, then one final implication follows: the pathway is not fixed throughout the life of the material. Cycling does not merely test stability. It progressively rewrites the route by which hydrogen exchange occurs. Early cycles may activate the surface, disrupt passivating barriers, redistribute catalysts, and open favorable interfaces. Intermediate cycles may benefit from defect-rich transport networks and interfacial continuity. Later cycles may become constrained by coarsening, contact loss, crack evolution, or structural relaxation of the very metastable states that enabled the earlier behavior. The route itself therefore has a history.

This view explains why cycling behavior in Mg-based hydrides is often nonmonotonic. Some materials improve before they degrade; others display outstanding initial kinetics that later become difficult to sustain. These trends are not always well described by the notion of one fixed mechanism that gradually slows down. More often, they indicate migration from one pathway to another as the material state evolves. A material that is initially surface-limited may become interface-assisted after activation. One that is initially defect-enabled may later become constrained by grain growth or interfacial relaxation. Reactive composite systems can likewise move between favorable and unfavorable routes as interfacial continuity or product distribution changes with repeated operation [39,40]. Cycle number should therefore be read not only as a durability count, but as a pathway-evolution index. For Mg-based hydrogen storage, the real challenge is not merely to create a favorable route once, but to preserve it as far as possible under repeated hydrogenation and dehydrogenation.

The argument of this section can therefore be condensed into one point. History matters because it determines where the reaction begins, which transport network hydrogen uses, what interfacial chemistry becomes active, and how all of these change during use. Catalysts, defects, interfaces, and metastable states are not separate categories whose effects are later added together. They are coupled means of writing, selecting, and stabilizing hydrogen sorption pathways. Once the problem is framed in this way, the transition to reproducibility becomes direct. If the field continues to compare materials mainly by formula while leaving pathway-defining states underdescribed, then reproducibility problems are not anomalous. They are inevitable.

## 5. Why Reproducibility Changes from Grams to Kilograms

### 5.1. Composition-Based Comparisons Fail When Reactive States Are Historically Non-Equivalent

If the preceding discussion is accepted, then the reproducibility problem in Mg-based hydrogen storage can no longer be treated as a narrow issue of instrumentation, operator discipline, or random experimental scatter. Those factors matter, but they are not the deepest source of difficulty. The central problem is that the field still tends to compare materials at the level of nominal composition while leaving the reactive state and the operative sorption pathway insufficiently defined. Once hydrogen sorption is viewed as state- and pathway-dependent, that habit becomes structurally inadequate. A material described as MgH_2_ with a given catalyst content may differ substantially from an apparently identical one in milling severity, passivation history, defect retention, surface accessibility, activation sequence, catalyst transformation state, cycle number, sample compaction, and thermal prehistory. If these variables are sufficient to change where hydrogen enters, which transport network it uses, or which interfacial route becomes operative, then the two materials are not truly the same in mechanistic terms, even if their formulas match. Under those conditions, imperfect reproducibility is not anomalous; it is exactly what should be expected.

This is why many disagreements in the Mg-based hydrogen storage literature should not first be interpreted as contradictions in chemistry. They are often contradictions in state description. Pressure–composition measurements, desorption profiles, onset temperatures, apparent activation energies, reversible capacities, and cycling-retention values are all influenced not only by composition but also by the pathway that is actually accessible under the chosen conditions. A plateau measured from a partially activated sample does not carry the same mechanistic meaning as one measured after a stable route has emerged. A desorption peak obtained from a defect-rich metastable state is not directly comparable to that of the same nominal material after partial relaxation. Capacity reported from an early-cycle pathway shaped by surface activation cannot be placed on the same footing as capacity measured after repeated cycling has already rewritten transport topology and interface continuity. Once the pathway perspective is adopted, it becomes clear that the field has often attempted to compare formulas while actually observing historically different mechanisms.

A recent MgH_2_ study [41] based on KH-modified TiO_2_/Nb_2_O_5_ and carbon-coated Ni nanoparticles provides a useful example of why composition-centered comparison is often mechanistically incomplete. At the nominal level, the system may be described rather simply as MgH_2_ modified by a catalyst prepared from KH, TiO_2_, Nb_2_O_5_, and C@Ni, all derived from commercially available precursors. However, the actual hydrogen-storage behavior was shown to depend strongly on the incorporation route rather than on catalyst identity alone. Two routes were compared: a one-step high-energy ball-milling method (BM), in which MgH_2_, the KH-modified oxide catalyst, and C@Ni were milled together, and a two-step route (MIX), in which MgH_2_ and the KH-modified oxide catalyst were first ball-milled and the C@Ni was subsequently incorporated by mixing without grinding bodies. The key point is that these two preparations generate nominally similar compositions but not the same reactive state. The resulting performance differences were substantial. The catalyst-containing materials lowered the dehydrogenation onset temperature from 321 °C for ball-milled MgH_2_ to below 236 °C, and the best-performing MIX sample reached an apparent activation energy of 93.8 kJ mol^−1^ compared with 152.5 kJ mol^−1^ for ball-milled MgH_2_. More importantly from the perspective of route sensitivity, the MgH_2_–1 wt% CAT–3 wt% C@Ni–MIX sample absorbed 5.77 wt% H_2_ at 150 °C in 50 min, 4.28 wt% at 100 °C in 120 min, and still 3.47 wt% at 75 °C in 120 min. These values were not matched by the corresponding BM compositions, even though the overall catalyst chemistry remained broadly comparable. The implication is that the measured hydrogen-storage behavior was not controlled by nominal composition alone, but by route-dependent differences in the accessible reactive state. The authors themselves made this point in a particularly useful way. SEM-EDS mapping confirmed overall compositional homogeneity and ruled out large-scale segregation, but it could not determine whether C@Ni nanoparticles were truly exposed at the outer surface or partially embedded within MgH_2_/CAT agglomerates, nor could it quantify the fraction of catalytically accessible Ni. On that basis, the paper explicitly argued that surface accessibility of C@Ni likely differentiates the BM and MIX routes and helps explain the superior low-temperature uptake of the MIX material. In the discussion, this distinction was developed into a direct process–structure–property interpretation: the incorporation route controls the near-surface availability of C@Ni adjacent to defect-rich (Ti/Nb)O_x_ domains, which in turn governs nucleation barriers, diffusion lengths, onset temperatures, apparent activation energies, and low-temperature hydrogenation behavior. This is exactly the kind of route-dependent hidden-variable problem that is often compressed away when materials are compared only by formula and additive loading.

Seen in this way, the mechanistic lesson of the case is not simply that KH-modified TiO_2_/Nb_2_O_5_ plus C@Ni is an effective catalyst system. The more important lesson is that a nominal catalyst system may exist in more than one operative state depending on how the catalyst is incorporated, how much of the active phase remains surface-accessible, what defect-rich oxide species are generated during processing, and how these features evolve during activation and cycling. A composition-centered reading would mainly compare catalyst identity and loading; a state-sensitive reading must additionally report incorporation route, post-processing surface accessibility, post-activation catalyst state, the pressure–temperature program under which low-temperature uptake was measured, and post-cycling morphological evolution. In this example, the route-dependent exposed state of Ni near defect-rich Ti/Nb suboxides is more mechanistically informative than catalyst identity alone. This is why nominally similar MgH_2_-based catalyst systems should not be treated as mechanistically equivalent unless the historically written reactive state has also been defined. This example shows why the reproducibility problem is often rooted less in nominal composition than in incomplete specification of route-written reactive state.

The consequence is not simply difficulty in repeating reported values. It is a deeper difficulty in interpreting what reported improvements actually mean. A catalyst may appear broadly effective when, in fact, its strongest benefit depends on a very specific activation sequence or interface geometry [41]. A nanostructure may seem intrinsically superior when the advantage actually derives from a retained metastable state that is difficult to preserve under different handling or cycling conditions. Some literature trends that look like universal composition effects are, on closer inspection, pathway effects tied to historically written state. Recognizing this does not diminish the value of the existing literature. It helps explain why a field so rich in promising results has nevertheless struggled to produce strongly cumulative comparisons. What has often been missing is not data, but a sufficiently explicit description of the reactive state that produced the data and the pathway that the data therefore represent. Reproducibility at this level should therefore be understood not simply as the ability to measure the same number twice, but as the ability to recreate a reactive state and, through that state, to recreate the corresponding sorption route.

### 5.2. Thermal Boundary Conditions Determine Whether a Favorable Pathway Can Be Expressed at Scale

**Figure 6:**
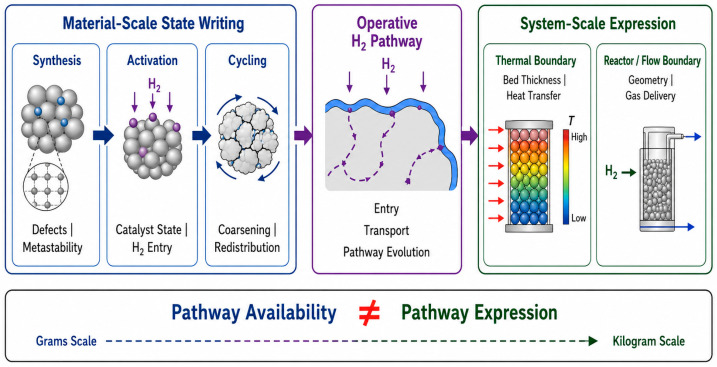
Relationship between material-scale reactive-state formation and system-scale pathway expression in Mg-based solid-state hydrogen storage. The operative H_2_-sorption pathway is first defined by synthesis-, activation-, and cycling-written states, but its practical expression at larger scale is further filtered by thermal and reactor/flow boundary conditions. The central message is that pathway availability at the material scale does not automatically translate into pathway expression at the system scale.

A favorable hydrogen-sorption pathway identified at laboratory scale does not automatically remain operative at tank scale. At the level of a few grams, material-internal variables such as interface accessibility, defect-assisted transport, catalyst state, and phase connectivity largely determine whether a pathway exists and how efficiently hydrogen can move through it. Once the same material is assembled into a larger storage body, however, the practical expression of that pathway becomes increasingly filtered by thermal and mass-transfer boundary conditions [42,43]. In that regime, heat transfer is not a peripheral engineering detail appended after the chemistry is solved. It becomes an external selector of whether an otherwise favorable internal pathway can still be sustained in practice. A pathway that is fully accessible in a small powder bed may become partially suppressed in a kilogram-scale system if heat cannot be supplied or removed rapidly enough, if hydrogen delivery becomes non-uniform through the bed, or if the reactive medium loses transport continuity under packing and confinement. This scale-translation problem is summarized in Figure 6. The figure distinguishes between material-scale state writing, where synthesis, activation, and cycling define whether a favorable hydrogen-sorption pathway becomes available, and system-scale expression, where thermal and flow boundary conditions determine whether that pathway can still operate effectively in a larger storage body. In this sense, the figure does not simply conclude the present discussion of reproducibility; it clarifies why a pathway identified at the grams scale may remain only partially expressed once heat transfer, bed geometry, and gas-delivery conditions become dominant constraints.

The importance of this distinction is especially clear for Mg-based systems because the same features that make them attractive for hydrogen storage also intensify their thermal-management burden. The relatively high stability of MgH_2_ means that hydrogen release remains strongly temperature-sensitive, while repeated absorption and desorption couple reaction progress to heat generation, heat consumption, volume change, and stress evolution within the hydride bed. Under such conditions, system performance depends not only on reversible capacity and local sorption kinetics, but also on how effectively hydrogen and heat can move through the storage body as a whole. System-level studies have repeatedly emphasized that container architecture, internal porous baffles, hydrogen-permeable channels, multi-scale porous matrices, fin-assisted heat exchange, and high-efficiency heat-conduction components such as heat pipes and heat pumps can all significantly affect the performance of solid-state hydrogen storage systems [42,43]. Likewise, monitoring temperature, pressure, flow rate, strain, and deformation is not simply an engineering afterthought. It is part of determining whether a favorable route remains accessible under realistic operating conditions.

The recent KH–TiO_2_–Nb_2_O_5_/C@Ni example reported by Ocampo et al. provides a particularly useful bridge between the laboratory and system scales [41]. In that study, the nominal catalyst chemistry could be described in a relatively simple way, yet the actual hydrogen-storage behavior depended strongly on the incorporation route. The authors showed that one-step BM and two-step MIX processing produced markedly different low-temperature hydrogenation responses, with the MIX sample reaching 5.77 wt% at 150 °C in 50 min, 4.28 wt% at 100 °C in 120 min, and still 3.47 wt% at 75 °C in 120 min. More importantly, they explicitly noted that SEM-EDS could confirm compositional homogeneity but could not determine whether C@Ni was truly exposed at the outer surface or partially embedded within MgH_2_/CAT agglomerates, and therefore could not quantify the fraction of catalytically accessible Ni. Their mechanistic interpretation was that route-dependent near-surface accessibility of Ni adjacent to defect-rich Ti/Nb suboxides controlled the density of exposed dissociation and spillover sites and thereby changed the practical uptake pathway. This is exactly the kind of state-sensitive difference that matters at laboratory scale. Yet even such a well-resolved pathway cannot be assumed to translate directly to larger storage bodies unless the associated thermal boundary conditions are also defined. A pathway may be available internally and still remain weakly expressed at scale if the heat-transfer environment prevents the material from accessing it uniformly or sustainably.

For this reason, laboratory-scale cycling should not be dismissed as irrelevant, but neither should it be overinterpreted as a direct proxy for storage-tank behavior. Laboratory-scale measurements remain indispensable because they are the scale at which hidden state variables, activation-written catalyst states, and route evolution can be identified with sufficient mechanistic resolution. Without such information, one cannot know which pathway the material is actually using. However, once the discussion turns to system translation, a second layer of description becomes essential. Sample mass, bed thickness, compact density, heating mode, wall contact, reactor geometry, pressure-flow program, and heat-transfer pathway must be reported alongside material-internal state variables if one wishes to judge whether a favorable pathway is merely available or can actually be expressed under realistic conditions [42,43]. In this sense, reproducibility at laboratory scale and reproducibility at system scale are nested rather than competing concepts. The first concerns whether a material-internal reactive state can be recreated. The second concerns whether the corresponding pathway can still operate when constrained by thermal and geometrical boundary conditions.

This distinction also changes how reproducibility should be interpreted in Mg-based hydrogen storage. Reproducing a number is not the same as reproducing a pathway. Two studies may report similar nominal compositions and even similar local kinetic metrics, yet differ substantially in the external conditions that determine whether a given route is thermally sustainable. Conversely, two samples may differ in their internally written reactive states but appear superficially similar when tested under boundary conditions that suppress those differences. This is why thermal boundary conditions should be treated as part of the minimum metadata set rather than as peripheral experimental detail [42,43]. If hidden state variables explain why nominally similar materials may not be mechanistically equivalent, then heat-transfer boundary conditions explain why nominally similar pathways may not remain equally expressible at larger scale. Once that is recognized, the transition to a metadata-aware, pathway-aware design framework becomes more direct. The field no longer needs only a better description of what material was tested. It also needs a better description of the external conditions under which a pathway was able—or unable—to operate.

## 6. Toward a Metadata-Aware, Pathway-Aware, and Boundary-Aware Design Framework

If the reproducibility problem is reformulated in this way, the need for a new design logic becomes unavoidable. The field does not need complexity for its own sake, nor does it need an ever-expanding vocabulary of loosely defined hidden factors. What it needs is a framework capable of describing what the reactive material actually is and under what conditions its operative hydrogen-sorption pathway can truly be expressed. In Mg-based hydrogen storage, the experimentally relevant object is not simply MgH_2_ with additive X or alloying element Y. It is MgH_2_ with additive X or alloying element Y in a historically written reactive state that enables, constrains, or suppresses a particular hydrogen-sorption pathway. Yet even this description is incomplete if it ends at the material scale. As Figure 6 now makes explicit, a favorable pathway may be available in a few-gram specimen but remain only partially expressed once the same material is transferred to a larger storage body in which bed thickness, packing state, wall contact, heating mode, reactor geometry, and gas-delivery conditions become active boundary constraints. A realistic design framework must therefore be not only metadata-aware and pathway-aware, but also boundary-aware, because pathway availability and pathway expression are related but not identical problems.

Such a framework does not need to be bureaucratic in order to be useful, but it does require sharper mechanistic discipline than is typical of composition-centered reporting. At minimum, the field should distinguish two coupled levels of description whenever a Mg-based material is reported to display a given performance advantage. The first concerns material-internal state definition: compositional identity, process history, activation history, and cycling history must be reported because they determine how the reactive state was written and which pathway became operative. The second concerns pathway-expression boundary conditions: sample mass, bed geometry, compact density, heating mode, reactor configuration, and gas-flow or pressure program must also be reported because they determine whether that pathway can still operate once the material is tested as more than an idealized small-scale powder. The first level explains why nominally similar materials may not be mechanistically equivalent; the second explains why nominally similar pathways may not remain equally expressible at larger scale. Once both levels are reported together, cumulative comparison becomes more realistic, because the field is no longer comparing formulas alone, nor abstract pathways alone, but historically written reactive states under defined internal and external constraints.

The practical value of this framework is that it changes how one interprets success. A strong result should not be read first as evidence that one additive, one nanostructure, or one synthesis label is universally superior. It should first be read as evidence that a particular historical route created a particular reactive state, and that this state enabled a particular pathway. This is a more demanding standard, but it is also a more scientifically useful one. It makes it easier to separate broadly transferable strategies from results that depend on narrow historical conditions. It also aligns laboratory-scale materials chemistry more closely with engineering relevance. Real hydride systems do not operate on abstract powders defined only by formula. They operate on materials with batch history, handling history, activation history, thermal history, and mechanical history, all of which influence whether a favorable route can be built and retained. From this perspective, metadata is not clerical overhead. It is the formal expression of material history.

A pathway-aware framework also changes how experiments should be organized. Under a conventional linear logic, synthesis produces a material, characterization identifies it, and testing ranks its performance. Under a state- and pathway-aware logic, these steps become a loop rather than a line. Synthesis writes the first state. Characterization verifies which hidden variables and metastable features were actually produced. Activation determines whether the intended catalytic and interfacial states emerge in operation. Sorption testing identifies which pathway is being accessed and whether that pathway survives cycling. Benchmarking then compares not only temperatures or capacities, but the success of route construction and route retention. This shift is important because it connects materials discovery with cumulative knowledge. A material is valuable not only if it yields a striking initial measurement, but if the favorable pathway responsible for that result can be intentionally created, independently recognized, and meaningfully preserved.

For Mg-based hydrogen storage, this reframing may be especially important because the field has already generated a large number of promising ideas. Catalysts, nanostructures, metastable hydrides, precursor routes, reactive composites, and interface engineering have all produced meaningful advances. What remains more difficult is determining which of these advances are fundamentally route-constructive in a robust sense and which depend on historically narrow conditions that are hard to reproduce or scale. A metadata-aware, pathway-aware framework does not resolve that problem automatically, but it supplies the right level of description at which the problem can be addressed. It clarifies what must be held constant, what must be reported, and what must be compared if Mg-based solid-state hydrogen storage is to move from a field rich in isolated successes toward one capable of more predictive design.

The central claim of this Perspective can therefore be stated in a compact form. Hydrogen sorption in Mg-based materials is not governed by nominal composition alone. It is governed by historically written reactive states that select, enable, and transform pathways of hydrogen exchange. Processing matters because it writes state. Metastability matters because it retains state. Catalysts and interfaces matter because they construct routes. Cycling matters because it rewrites routes. Reproducibility becomes difficult when pathway-defining states remain underdescribed, and materials design becomes more cumulative when those states are made explicit through a metadata-aware framework. From that perspective, the future of Mg-based solid-state hydrogen storage depends not only on better ingredients, but on better-defined, better-constructed, and better-preserved pathways.

## Figures and Tables

**Table 1:** Hidden variables in Mg-based hydrogen storage: when they are written, how they can be measured, and what should be minimally reported.

Hidden Variable	Main Stage Where It Is Written	Typical Measurable Proxy	Priority Characterization	Mechanistic Consequence for Sorption Behavior	Minimum Reporting Item
Particle/agglomerate size distribution	Synthesis/milling	d50, d90, agglomerate size, distribution width	SEM, TEM, laser particle sizing	Changes hydrogen-entry area, local diffusion length, and contact continuity	Starting and final particle/agglomerate size, and whether the value refers to primary particles or agglomerates
Defect density/microstrain	Synthesis/deformation/high-energy processing	Peak broadening, Williamson–Hall strain, defect-sensitive spectral features	XRD refinement, TEM, EPR or positron methods when available	Changes nucleation barrier, diffusion pathway selection, and local reactivity	At least one defect-sensitive proxy after synthesis and before hydrogen testing
Surface/passivation state	Synthesis + handling	Oxide thickness, surface oxygen state, passivation history	XPS, surface-sensitive TEM, controlled air-exposure record	Changes the initial hydrogen-entry barrier and early activation behavior	Passivation/air-exposure protocol plus at least one surface-chemistry result
Amorphous or metastable phase fraction	Synthesis	Amorphous halo, minor metastable peaks, local diffraction evidence	XRD, TEM/SAED	Changes local energetics and preserves historically written reactive states	State whether metastable/amorphous features were checked before performance testing
Catalyst state after activation	First hydrogenation/dehydrogenation	Oxidation-state change, interfacial transformation, in situ generated phase	Post-activation XPS, XRD, TEM	Determines the real active state rather than the nominal additive identity	Full first-cycle activation routine plus at least one post-activation catalyst-state characterization
Interfacial contact topology/exposed active-site accessibility	Activation + incorporation route	Exposed vs. embedded catalyst fraction, contact geometry, surface accessibility	SEM/TEM plus surface-sensitive analysis	Controls whether catalytic sites are actually available for hydrogen dissociation and transfer	Incorporation route and evidence showing whether the active phase is merely dispersed or surface-accessible
Cycle index/cycle prehistory	Cycling	First-cycle, activated-cycle, stabilized-cycle, late-cycle state	Explicit reporting in sorption datasets	Early-cycle and steady-cycle pathways may differ substantially	Every kinetic/capacity dataset should state cycle number and prehistory
Microstructural evolution during cycling	Cycling	Crack formation, coarsening, agglomeration, catalyst redistribution	Pre-/post-cycling SEM, TEM, XRD	Changes transport continuity and route retention during repeated use	At least one post-cycling structural comparison linked to the reported cycling data
Sample mass/bed geometry/compact density	Testing	Mass, bed thickness, pellet vs. loose powder, packing density	Direct reporting	Changes transport length, contact state, and scale of thermal gradients	Sample mass and physical configuration must be reported for every key dataset
Thermal boundary condition/gas-program boundary	Testing/scale translation	Heating mode, heating rate, reactor geometry, pressure ramp, dwell time	Direct reporting	Determines whether a favorable internal pathway can actually be expressed at scale	Heating/cooling protocol, pressure–temperature program, and basic reactor boundary conditions

## References

[1] Schlapbach L., Züttel A. (2001). Hydrogen-storage materials for mobile applications. Nature.

[2] Sakintuna B., Lamari-Darkrim F., Hirscher M. (2007). Metal hydride materials for solid hydrogen storage: A review. Int. J. Hydrogen Energy.

[3] Züttel A. (2003). Materials for hydrogen storage. Mater. Today.

[4] Jain I.P., Lal C., Jain A. (2010). Hydrogen storage in Mg: A most promising material. Int. J. Hydrogen Energy.

[5] Xu Y., Zhou Y., Li Y., Hao Y., Wu P., Ding Z. (2024). Magnesium-Based Hydrogen Storage Alloys: Advances, Strategies, and Future Outlook for Clean Energy Applications. Molecules.

[6] Zaluska A., Zaluski L., Ström-Olsen J.O. (1999). Nanocrystalline magnesium for hydrogen storage. J. Alloys Compd..

[7] Huot J., Liang G., Boily S., Van Neste A., Schulz R. (1999). Structural study and hydrogen sorption kinetics of ball-milled magnesium hydride. J. Alloys Compd..

[8] Liang G., Huot J., Boily S., Van Neste A., Schulz R. (1999). Catalytic effect of transition metals on hydrogen sorption in nanocrystalline ball-milled MgH_2_–Tm (Tm = Ti, V, Mn, Fe and Ni) systems. J. Alloys Compd..

[9] Oelerich W., Klassen T., Bormann R. (2001). Metal oxides as catalysts for improved hydrogen sorption in nanocrystalline Mg-based materials. J. Alloys Compd..

[10] Barkhordarian G., Klassen T., Bormann R. (2003). Fast hydrogen sorption kinetics of nanocrystalline Mg using Nb_2_O_5_ as catalyst. Scr. Mater..

[11] Suryanarayana C. (2001). Mechanical alloying and milling. Prog. Mater. Sci..

[12] Fecht H.-J. (1995). Nanostructure formation by mechanical attrition. Nanostruct. Mater..

[13] de Jongh P.E., Adelhelm P. (2010). Nanosizing and nanoconfinement: New strategies towards meeting hydrogen storage goals. ChemSusChem.

[14] Sadhasivam T., Kim H.-T., Jung S., Roh S.-H., Park J.-H., Jung H.-Y. (2017). Dimensional effects of nanostructured Mg/MgH2 for hydrogen storage applications: A review. Renew. Sustain. Energy Rev..

[15] Xu Y., Li Y., Hou Q., Hao Y., Ding Z. (2024). Ball Milling Innovations Advance Mg-Based Hydrogen Storage Materials Towards Practical Applications. Materials.

[16] Wagemans R.W.P., van Lenthe J.H., de Jongh P.E., van Dillen A.J., de Jong K.P. (2005). Hydrogen storage in magnesium clusters: Quantum chemical study. J. Am. Chem. Soc..

[17] Li W., Li C., Ma H., Chen J. (2007). Magnesium nanowires: Enhanced kinetics for hydrogen absorption and desorption. J. Am. Chem. Soc..

[18] Nielsen T.K., Manickam K., Hirscher M., Besenbacher F., Jensen T.R. (2009). Confinement of MgH_2_ nanoclusters within nanoporous aerogel scaffold materials. ACS Nano.

[19] Bortz M., Bertheville B., Böttger G., Yvon K. (1999). Structure of the high pressure phase γ-MgH_2_ by neutron powder diffraction. J. Alloys Compd..

[20] Jeon K.-J., Moon H.R., Ruminski A.M., Jiang B., Kisielowski C., Bardhan R., Urban J.J. (2011). Air-stable magnesium nanocomposites provide rapid and high-capacity hydrogen storage without using heavy-metal catalysts. Nat. Mater..

[21] Floriano R., Leiva D.R., Deledda S., Hauback B.C., Botta W.J. (2013). Nanostructured MgH_2_ obtained by cold rolling combined with short-time high-energy ball milling. Mater. Res..

[22] El-Eskandarany M.S., Banyan M., Al-Ajmi F. (2018). Discovering a new MgH_2_ metastable phase. RSC Adv..

[23] Reilly J.J., Wiswall R.H. (1968). Reaction of hydrogen with alloys of magnesium and nickel and the formation of Mg_2_NiH_4_. Inorg. Chem..

[24] Bobet J.-L., Akiba E., Nakamura Y., Darriet B. (2000). Study of Mg–M (M = Co, Ni and Fe) mixtures elaborated by reactive mechanical alloying: Hydrogen sorption properties. Int. J. Hydrogen Energy.

[25] Olmez R., Çakmak G., Oztürk T. (2010). Combinatorial search for hydrogen storage alloys: Mg–Ni and Mg–Ni–Ti. Int. J. Hydrogen Energy.

[26] Cho Y.H., Aminorroaya S., Liu H.K., Dahle A.K. (2011). The effect of transition metals on hydrogen migration and catalysis in cast Mg–Ni alloys. Int. J. Hydrogen Energy.

[27] Zeng K., Klassen T., Oelerich W., Bormann R. (1999). Thermodynamic analysis of the hydriding process of Mg–Ni alloys. J. Alloys Compd..

[28] Li Q., Lu Y., Luo Q., Yang X., Yang Y., Tan J., Dong Z., Dang J., Li J., Chen Y. (2021). Thermodynamics and kinetics of hydriding and dehydriding reactions in Mg-based hydrogen storage materials. J. Magnes. Alloys.

[29] Zhou C., Hu C., Li Y., Zhang Q. (2020). Crystallite growth characteristics of Mg during hydrogen desorption of MgH_2_. Prog. Nat. Sci. Mater. Int..

[30] Nogita K., Tran X.Q., Yamamoto T., Tanaka E., McDonald S.D., Gourlay C.M., Yasuda K., Matsumura S. (2015). Evidence of the hydrogen release mechanism in bulk MgH_2_. Sci. Rep..

[31] Patelli N., Migliori A., Morandi V., Pasquini L. (2020). Interfaces within biphasic nanoparticles give a boost to magnesium-based hydrogen storage. Nano Energy.

[32] Zhang J., Liu H., Sun P., Guo X., Zhou C., Fang Z.Z. (2022). The effects of crystalline defects on hydrogen absorption kinetics of catalyzed MgH_2_ at ambient conditions. J. Alloys Compd..

[33] Duan C., Wang X., Wang H., Wu M., Fan Y., Wu J., Qu T., Liu B., Hu L., Liang P. (2024). The impact of vacancy defective MgH_2_ (001)/(110) surface on the dehydrogenation of MgH_2_@Ni-CNTs: A mechanistic investigation. J. Mater. Sci. Technol..

[34] Yang W.N., Shang C.X., Guo Z.X. (2010). Site density effect of Ni particles on hydrogen desorption of MgH_2_. Int. J. Hydrogen Energy.

[35] Yang X., Hou Q., Yu L., Zhang J. (2021). Improvement of the hydrogen storage characteristics of MgH_2_ with a flake Ni nano-catalyst composite. Dalton Trans..

[36] Si T.-Z., Zhang X.-Y., Feng J.-J., Ding X.-L., Li Y.-T. (2021). Enhancing hydrogen sorption in MgH_2_ by controlling particle size and contact of Ni catalysts. Rare Met..

[37] Zhang J., He L., Yao Y., Zhou X., Yu L., Lu X., Zhou D. (2020). Catalytic effect and mechanism of NiCu solid solutions on hydrogen storage properties of MgH_2_. Renew. Energy.

[38] Zhang J., Hou Q., Liu Y., Yang X. (2023). A fancy hydrangea shape bimetallic Ni–Mo oxide of remarkable catalytic effect for hydrogen storage of MgH_2_. J. Ind. Eng. Chem..

[39] Ding Z., Lu Y., Li L., Shaw L. (2019). High reversible capacity hydrogen storage through Nano-LiBH_4_ + Nano-MgH_2_ system. Energy Storage Mater..

[40] Ding Z., Li H., Shaw L.L. (2020). New insights into the solid-state hydrogen storage of nanostructured LiBH4–MgH2 system. Chem. Eng. J..

[41] Ocampo R.A., Arias Velandia J., Lenis J.A., Zuleta Gil A.A., Bello S., Correa E., Arrieta C., Bolívar F.J., Echeverria Echeverria F. (2026). Enhanced hydrogen storage properties of magnesium hydride using a KH-TiO2-Nb2O5/carbon-coated nickel nanoparticles catalyst. J. Alloys Compd..

[42] Lototskyy M.V., Tolj I., Pickering L., Sita C., Barbir F., Yartys V.A. (2017). The use of metal hydrides in fuel cell applications. Prog. Nat. Sci. Mater. Int..

[43] Liu J., Sun L., Yang J., Guo D., Chen D., Yang L., Xiao P. (2022). Ti–Mn hydrogen storage alloys: From properties to applications. RSC Adv..

